# Expression of Concern: Knockdown of Tcf3 enhances the wound healing effect of bone marrow mesenchymal stem cells in rat

**DOI:** 10.1042/BSR-2018-0369_EOC

**Published:** 2023-02-09

**Keywords:** bone marrow mesenchymal stem cells, rat, Tcf3, wound healing

The Editorial Office has been made aware of potential issues surrounding the scientific validity of this paper, hence has issued an expression of concern to notify readers whilst the Editorial Office investigates. The Figure 2D Si-Tcf3#3 image seems to appear as the Figure 3C Inhibitor NC panel from Zhao et al. 2017 (doi: 10.1042/bsr20170837) and as the 143B/sh-VAV3+inhibitor panel of Figure 6E from Xiao et al. 2020 (doi: 10.18632/aging.103762). In addition, the Figure 2D Si-Tcf3#1 image seems to appear as the Figure 2C sh-NC panel of Bian 2020 (doi: 10.2147/OTT.S180850), and the Figure 4G CK-18 and GAPDH western blots also seem to appear as the Figure 6A CaBP-9k bands and the 2C ER-beta bands respectively from Liu et al. 2020 (doi: 10.1042/bsr20191330).

